# Oxymatrine alleviates cognitive dysfunction in mice caused by chronic cerebral hypoperfusion by promoting SIRT1/ PINK1-mediated mitophagy

**DOI:** 10.3389/fphar.2026.1736022

**Published:** 2026-04-24

**Authors:** Beier Tong, Xufeng Li, Wangwang Hu, Beilei Ding, Jie Chen, Jiaoni Gong, Lei Gu, Lu Shi

**Affiliations:** 1 Department of Rehabilitation Medicine, Ningbo Medical Center Lihuili Hospital, Ningbo, Zhejiang, China; 2 Department of Orthopedic Surgery Medicine, Ningbo Medical Center Lihuili Hospital, Ningbo, Zhejiang, China

**Keywords:** chronic cerebral hypoperfusion, cognitive dysfunction, mitophagy, oxymatrine, PINK1

## Abstract

Oxymatrine (OMT) has been reported to exert neuroprotective effects in cerebral ischemia. Mitochondrial dysfunction is closely associated with the neurological deficits caused by chronic cerebral hypoperfusion (CCH). However, the potential role of mitochondrial protection in the neuroprotective effects of OMT, as well as the underlying mechanisms, remains to be fully elucidated. The present study aimed to investigate the mechanistic basis of OMT-mediated neuroprotection *in vivo* using a mouse model of CCH-induced cognitive impairment established by bilateral common carotid artery stenosis (BCAS), and *in vitro* using an HT22 cell injury model induced by oxygen-glucose deprivation/reperfusion (OGD/R). The *in vivo* results showed that OMT effectively alleviated CCH-induced cognitive impairment in mice. In addition, OMT treatment attenuated OGD/R-induced neuronal injury in HT22 cells. Mechanistically, OMT activated SIRT1 and promoted PINK1/Parkin-mediated mitophagy in neurons. Moreover, pharmacological inhibition of SIRT1 suppressed OMT-induced activation of PINK1/Parkin-mediated mitophagy, as well as the recovery of neuronal and cognitive function in both the cell and mouse models. These findings suggest that OMT enhances mitophagy through modulation of SIRT1, thereby ameliorating neuronal dysfunction and cognitive deficits associated with CCH and OGD/R. This study provides preclinical mechanistic evidence supporting further investigation of OMT in chronic cerebral hypoperfusion-related cognitive impairment.

## 1 Introduction

The global aging population and the absence of effective treatments for dementia have led to an increase in its prevalence, significantly affecting elderly health and quality of life. Alzheimer’s disease (AD) and vascular dementia (VaD) are the two most common forms of dementia in the elderly, and the number of VaD cases is projected to reach 10 million by 2030 (Cumming and Brodtmann, 2010). VaD significantly reduces quality of life, imposing substantial medical and economic burdens on families and society (Wolters and Ikram, 2019). Chronic cerebral hypoperfusion (CCH), a key pathophysiological factor in both AD and VaD, contributes to blood-brain barrier (BBB) disruption and chronic neuroinflammation (Ashraf et al., 2016; Iturria-Medina et al., 2016). CCH results from sustained low blood supply to the brain, causing endothelial injury, BBB disruption, and increased permeability (Livingston et al., 2020; Nation et al., 2019). This allows neurotoxic substances and pathogens to penetrate the brain, triggering immune responses, oxidative stress, and neuroinflammation, ultimately leading to neuronal damage, cognitive decline, and dementia (De Silva and Faraci, 2016). Despite progress, the exact mechanisms linking cerebrovascular dysfunction to cognitive dysfunction remain unclear. Current treatments focus on symptom management and risk factor control but are limited by modest efficacy and significant side effects (Wu et al., 2012). There is an urgent need to identify new therapeutic targets and drugs for VaD.

Mitochondria are essential for energy production, metabolism, and signal transduction, with stable mitochondrial function being critical for cell survival, particularly in energy-demanding neurons (Mishra and Thakur, 2023). Mitochondrial damage is a hallmark of VaD (Ma et al., 2013). In neurodegenerative diseases, damaged mitochondria accumulate in neurons, resulting in bioenergy deficits, impaired cellular signaling, and disrupted calcium buffering, among other pathological changes (Shafique et al., 2023). Mitochondrial quality control mechanisms, including mitophagy, are vital for maintaining mitochondrial health and function. Mitophagy, the process by which defective mitochondria are transported to lysosomes for degradation, is crucial in preventing mitochondrial accumulation. A deficiency in mitophagy leads to cellular dysfunction and/or death, contributing to the pathology of neurodegenerative diseases (Li et al., 2021; Mani et al., 2021; Martinez-Vicente, 2017). The PINK1/Parkin-dependent mitophagy pathway is a key focus of research in mammalian cells. PINK1 and Parkin mediate mitochondrial damage recognition through the ubiquitination of defective mitochondria (Li et al., 2023), followed by the recruitment of autophagy receptors that guide the formation of autophagosomes around the ubiquitinated mitochondria, ultimately clearing them via autophagic and lysosomal pathways (Lazarou et al., 2015; Lou et al., 2020). Evidence suggests that upregulation of mitophagy has therapeutic potential for VaD (Zheng et al., 2021), and alterations in the PINK1/Parkin pathway may contribute to the pathogenesis of neurodegenerative diseases such as Alzheimer’s Disease (Cai and Jeong, 2020; Fang et al., 2019; Ye et al., 2015). PINK1-mediated phosphorylation of Parkin facilitates the recognition of damaged mitochondria by autophagosomes, promoting their degradation in lysosomes, reducing oxidative stress, protecting hippocampal neurons, and improving cognitive function (Tanaka, 2020; Quinn et al., 2020).

Traditional Chinese Medicine (TCM) is now recognized by the World Health Organization (WHO) in its International Classification of Diseases and is increasingly accepted globally as complementary medicine (Cyranoski, 2018). Oxymatrine (OMT), a quinolone alkaloid extracted from *Sophora flavescens Aiton (*Fabaceae*)*, is a major pharmacologically active metabolite known for its properties in clearing heat, eliminating dampness, killing parasites, and promoting diuresis (Jiang et al., 2018; He et al., 2015). Growing evidence supports the therapeutic potential of OMT *in vitro* and *in vivo*, with pharmacological activities including anti-inflammatory, analgesic, antioxidative, anti-fibrotic, anti-apoptotic, and anti-tumor effects (Lan et al., 2020). Studies have demonstrated OMT’s neuroprotective potential, particularly in neurological diseases (Dhurandhar et al., 2024). OMT has been shown to inhibit neuroinflammation by modulating microglial M1/M2 polarization via the TLR4/NF-κB signaling pathway (Wang et al., 2021). Recent research indicates that OMT also protects against hypoxic-ischemic brain injury in neonatal mice by activating the Wnt/β-catenin pathway (Lan et al., 2023), and in neonatal rats via the PI3K/Akt/GSK3β/mTOR pathway (Wei et al., 2021; Liu et al., 2020). However, the therapeutic potential and underlying mechanisms of OMT in the treatment of CCH-induced cognitive disorders remain unclear.

This study investigates the protective effects of OMT pretreatment in a chronic cerebral hypoperfusion model induced by BCAS in mice, focusing on the mechanism of mitophagy mediated through the PINK1/Parkin pathway. The findings aim to enhance our understanding of OMT’s potential as a therapeutic approach for VaD.

## 2 Methods

### 2.1 Chemicals and reagents

HT22 cells (mouse hippocampal neurons, No. CL-0697) were acquired from Wuhan Procell Life Science and Technology Co., LTD. Antibodies for PTEN-induced kinase 1 (PINK1, 23274-1-AP) and parkinson disease protein 2 (PARKIN, 14060-1-AP) came from Proteintech Group Inc. (Wuhan, China). The antibodies of microtubule-associated protein 2 (MAP2, A2572), postsynaptic density protein-95 (PSD95, A6194), synaptophysin (SYN, A6344) and sirtuin 1 (SIRT1, A11267) were sourced from Abclonal Technology Co., Ltd., Wuhan, China. Reverse transcription and real-time quantitative polymerase-chain reaction (RT-qPCR) kits were purchased from Takara Biotechnology Co., Ltd. (Dalian, China). The OMT (HY-N0158, purity 99.92%, research grade), Mdivi-1 (HY-15886) and EX (HY-15452) were obtained from MedChemExpress Co., Ltd. (Shanghai, China). Isoflurane was obtained from Baxter Healthcare Co. (Deerfield, IL, USA). RIPA lysis buffer was obtained from Beyotime co., Ltd. (Shanghai, China). Tetrechloro-tetraethylbenzimidazol carbocyanine iodide (JC-1) was purchased from Beyotime (China). MitoTracker and LysoTracker were obtained from Thermo Fisher Scientific (America). All oligonucleotide primers were synthesized by Qinke Biotech Co., Ltd. (Wuhan, China). The reagents used in other experiments were all of analytical grade.

### 2.2 Animals and treatments

The mouse CCH model was constructed by using the BCAS method. SPF C57BL/6 mice weighing 25 ± 5 g (males) were obtained and handled in the Hubei Provincial Center for Disease Control and Prevention. All animal experiments followed the National Research Council’s Guide for the Care and Use of Laboratory Animals. The principles of the 3Rs were applied to minimize unnecessary harm to the animals. OMT doses were selected based on previous studies reporting neuroprotective effects of OMT in experimental cerebral ischemic/hypoxic injury models and two doses (30 and 60 mg/kg) were used to evaluate dose responsiveness (Zhao et al., 2015; Huang et al., 2012; Dong et al., 2019). After all the experimental animals were transported to the experimental center and acclimatized for 7 days, surgical and therapeutic procedures were carried out. 30 mice were randomly divided into five group (n = 6): sham operation group (Control), CCH group (CCH), CCH + OMT medium-dose (30 mg/kg) group, CCH + OMT high-dose (60 mg/kg) group and CCH + OMT high-dose + SIRT1 intervention group (EX527) group. After anesthetizing the mice, the bilateral common carotid artery sheaths were fully exposed. The nerves and fascia surrounding the carotid artery were carefully separated using ophthalmic forceps. Then microcoils were twined by rotating it around each common carotid artery were used. Finally, the anatomical positions of each layer of tissue were restored layer by layer. The method on the other side is the same as this. After the ligation of the bilateral common carotid arteries was completed, the incisions were sutured. The sham operation group had the same operations as the CCH group except that the common carotid artery was not operated. In the OMT group, the drug was administered continuously for 3 days every 12 h by intraperitoneal injection. The sham operation group and the CCH group were injected with normal saline. EX527 (5 mg/kg/ day) was intraperitoneally injected 0.5 h before OMT administration. On the fifth day after surgery, behavioral tests were conducted. The order of the tests from low to high was open field test (OFT), novel objection recognition test (NOR), and Y-maze test. After behavioral testing, one hemisphere was used for immunofluorescence and the hippocampus from the other hemisphere was used for biochemical analyses.

### 2.3 Behavior tests

#### 2.3.1 NOR test

On the first day (adaptation stage), no objects should be placed in the test box. Place the mice with their backs facing the box wall into the test box and let them move freely for 10 min, then put them back into the original breeding cage. On the second day (the testing phase), two identical objects (AA) are symmetrically placed in the test box (9 cm on the long axis and 10 cm on the short axis). The mice were placed in the test box from the same distance point between two objects, moved freely for 10 min, and then put back into the original cage. One hour later, two different objects (A and B) were placed at the same positions as mentioned above, and then the mice were put into the test chamber in the same way and moved freely for 5 min, allowing them to freely explore the objects in the test chamber. When different objects (A and B) are placed, a stopwatch is used to record the exploration time of the mouse on the old object (A) and the new object (B), that is, the time spent interacting with the objects. Interaction is defined as direct contact with an object, including touching it with the mouth, nose or PAWS. Accidental contact (i.e., hitting an object during movement) is regarded as no interaction. Calculate the discrimination index (DI) for exploring novel objects.

#### 2.3.2 OFT test

The same behavioral arena was used for both the OFT and NOR test. A 40W incandescent lightbulb placed 2.8 m above the field’s center was used to illuminate the OFT. After 3 min of acclimatization, the mice underwent a 5-min test. Every mouse to mouse, the device was cleaned with 75% ethanol. The mice’s exploratory route, the total distance traveled, the distance traveled inside the core region, and the amount of time spent were all recorded and analyzed.

#### 2.3.3 Y-maze test

The maze has three arms (30 × 8 × 20 cm in size), and the Angle between each arm is 120°. Randomly designate the three arms as the starting arm (always open), the familiar arm (always open), and the new arm (blocked during the training phase and open during the test phase). In the first stage, place the mouse at the end of the starting arm, facing the wall and away from the center, and allow the two open arms to freely explore for 5 min. After a delay of 2 h, open the new arm and conduct the test. At this stage, the mice were placed in the same starting arm and were allowed to enter all three arms of the maze for 5 min. Record the central trajectory and the total time spent exploring each arm. The preference index is defined as the percentage of time spent exploring a new group divided by the total time spent in the new group and the familiar group.

All test trials were recorded and analyzed by the SMART video tracking system (SMART v3.0, Panlab, Spain).

### 2.4 Transmission electron microscope

The hippocampal tissues were isolated and extracted, pre-fixed in 4% paraformaldehyde (pH 7.4) containing 2.5% glutaraldehyde, and then fixed in 0.1 M sodium bicarbonate buffered 1% OsO4 solution. After dehydration with a stepwise ethanol gradient solution, the sample was incubated with propylene oxide, and then impregnated and embedded in Spurr resin with a mixture of propylene oxide/Surr (1:1). The ultrastructure of mitochondria was observed by transmission electron microscopy.

### 2.5 Immunofluorescence staining

The tissue sections were dewaxed in xylene and then hydrated. Then boil in citric acid buffer (10 mM, pH 6.0) for 3 min and rinse three times with PBS. After blocking 5% BSA for 20 min, incubate overnight with the primary antibody at 4 °C. Tyramide signal amplification (TSA; Recordbio, China) was applied for 10 min, then incubated with fluorescent secondary antibody (1:200) at room temperature for 2 h, and stained with DAPI (1 μg/ml) for 15 min. Fluorescence images were collected using an inverted microscope.

### 2.6 Western blot

The total protein of the sample to be tested was extracted with RIPA lysis buffer, and the protein concentration was detected by BCA protein assay. The protein samples (40 μg) were fractionated and separated using 10% sodium dodecyl sulfate-polyacrylamide gel, transferred onto PVDF membranes, and sealed in 5% skimmed milk at room temperature for 2 h. Next, place the membrane in the corresponding primary antibody (MAP2 (1:1000), PSD95 (1:1000), GAPDH (1:5000), SYN (1:1000), PINK1 (1:1000), and PARKIN (1:1000)) and incubate it overnight at 4 °C. Wash three times with TBST and incubate in the corresponding secondary antibody for 30 min. The immunoblotting bands were obtained through development and exposure using the ECL chemiluminescence detection kit. The protein bands were analyzed using ImageJ software.

### 2.7 RT-PCR

Take fresh hippocampal tissue or cells and add them to the lysis buffer before using a homogenizer. Accurately estimate the volume of the supernatant of the lysate, add an equal volume of 70% ethanol, and immediately pipette to mix well. Immediately add the mixture to an adsorption column RA, centrifuge at 13,000 rpm for 30 s, and discard the waste liquid. Add 700 μL of deproteinized solution RW1, let it stand at room temperature for 30 s, centrifuge at 13,000 rpm for 30 s, and discard the waste liquid. Add 500 μL of rinsing solution RW, centrifuge at 13,000 rpm for 30 s, and discard the waste liquid. Add 500 μL of rinsing solution RW and repeat once. Return the adsorption column RA to the empty collection tube and centrifuge at 13,000 rpm for 2 min to remove the rinsing solution as much as possible. Remove the adsorption column RA and place it in a clean 1.5 mL centrifuge tube. Add 30–50 μL of RNase free water to the middle part of the adsorption membrane according to the expected RNA yield. Let it stand at room temperature for 1 min and centrifuge at 13,000 rpm for 1 min to obtain the RNA solution. Take 1 μL of the solution and measure OD260 and OD280 with a micro spectrophotometer. Calculate the OD260/OD280 value, calculate the concentration and purity of RNA, and estimate the RNA mass based on this. According to the concentration of RNA in the samples, dilute each sample RNA with DEPC water to 1000 ng/μL. Place the labeled 200 μL enzyme-free EP tubes on ice and use the reverse transcription kit to obtain cDNA products. Store them in a −80 °C refrigerator or immediately use them for qPCR reactions. Real-time fluorescence quantitative PCR detection was conducted, reaction conditions were set, dissolution curves were plotted, and the relative expression of the target gene was calculated using the 2^−ΔΔCT^ method. The primer sequences are shown in Table 1.

**TABLE 1:** Oligonucleotide primer sequences for real-time quantitative PCR.

Genes	Forward primer sequences (5'→3′)	Reverse primer sequences (5'→3′)
GAPDH	TGA​AGG​GTG​GAG​CCA​AAA​G	AGT​CTT​CTG​GGT​GGC​AGT​GAT
MAP2	AGG​TCA​GAA​CCA​ATT​CGC​AGA	ACT​TTG​GAG​GAG​TGC​GGA​TG
SYN	ACC​TCG​GTG​GTG​TTT​GGC​TT	TGCCCGTAATCGGGTTGA
PSD95	GCA​GGT​TGC​AGA​TCG​GAG​AC	ACT​GAT​CTC​ATT​GTC​CAG​GTG​CT
PINK1	CTG​CCT​GAG​ATG​CCT​GAG​TC	GTG​CAG​ACG​GTC​TCT​TGC​T
PARKIN	GGA​GGA​GGC​CTG​GAT​GAC​TA	ACA​AAC​ACT​ATC​ATG​GTC​ACC​G
SIRT1	TCT​GAA​AGT​GAG​ACC​AGT​AGC	ATA​ACA​TCG​CAG​TCT​CCA​AGG

MAP2, microtubule-associated protein 2; PSD95, postsynaptic density protein-95; SYN, synaptophysin; PINK1, PTEN-induced kinase 1; PARKIN, parkinson disease protein 2; SIRT1, Sirtuin 1.

### 2.8 Culture and differentiation of HT22

Simulate the CCH environment using the oxygen and glucose deprivation and reperfusion (OGD/R) model. On the seventh day of routine culture of hippocampal neurons, the culture medium was discarded and washed three times with preheated EBSS solution to remove any remaining culture medium. Add the same volume of EBSS solution as the original culture medium to simulate a sugar-deficient environment. The cell culture dishes were placed in an hypoxia chamber with a temperature controlled at 37 °C for 6 h to simulate the hypoxic environment in the body. After 6 h, remove the culture dish, discard the EBSS solution, and replace it with normal NB culture medium or drug-containing culture medium. Then continue to culture in a CO_2_ incubator for 24 h. Meanwhile, the cells were treated with 30 and 60 μM OMT. SIRT1 and EX527 inhibitors and mitophagy inhibitor Mdivi-1 were treated 24 h before OMT treatment.

### 2.9 Mitochondrial and lysosome colocalization detection

The HT22 in the confocal dish was washed with PBS after the culture medium was removed. Then, incubate with DMEM/F12 containing MitoTracker (1:1000) and LysoTracker (1:1000) at 37 °C for 20 min, followed by three PBS washes. The cell nuclei were stained with Hoechst (1:1000) for 20 min and then washed three times. Observe the cells with a confocal microscope.

### 2.10 Detection of intracellular ROS

To measure intracellular ROS, we used the detectable 2′,7′-dichlorofluorescin diacetate (DCFH-DA) fluorescent substance. On a 6-well cell culture plate, we seeded the cells at a density of 3 × 10^4^ per well for 24 h. OMT were pretreated and cells were incubated with 20 μM DCFH-DA in PBS solution for 30 more minutes. Subsequently, the cells were observed using a fluorescence microscope (Nikon Eclipse Ti2, Tokyo, Japan) and images were captured.

### 2.11 Detection of mitochondrial membrane potential

Prepare the JC-1 staining working solution. Wash the cells three times with PBS, then add 1 mL of culture medium and JC-1 staining working solution each time, mix thoroughly and incubate in a CO2 incubator for 20 min. After incubation, discard the supernatant and wash the cells twice with pre-cooled 1 × JC-1 staining buffer. Add 2 mL of cell culture medium to each well, observe and collect slides under a fluorescence microscope, and then analyze and calculate the fluorescence intensity of cells in each group.

### 2.12 Statistical methods

Statistical analysis and chart drawing were performed using SPSS 26.0 and GraphPad Prism 8.0 software. The data were presented as mean ± standard error of the mean (SEM). Normality was tested using the Shapiro-Wilk test. For comparisons between two groups, if the data met the normality assumption, an unpaired t-test was used. For comparisons among multiple groups, a one-way analysis of variance (ANOVA) was employed, followed by Tukey’s multiple comparison test. If the data did not meet the normality assumption, the Kruskal–Wallis H test was used, and pairwise comparisons were made using the Nemenyi test. A p value <0.05 was considered statistically significant.

## 3 Results

### 3.1 OMT improved the cognition and spatial memory behaviors changes of CCH mice

**FIGURE 1:**
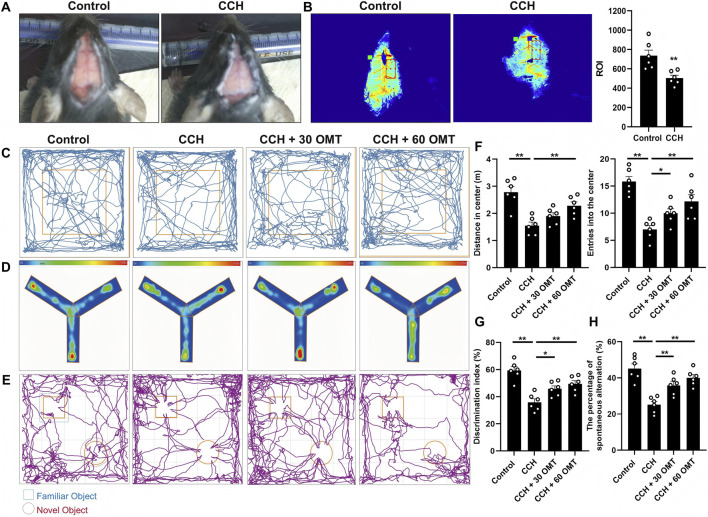
OMT improved the cognition and spatial memory behaviors changes of CCH mice. **(A)** General view of cerebral blood supply in mice after BCAS modeling; **(B)** Laser speckle imaging and quantitative analysis of cerebral blood flow in CCH mice; **(C,F)** The representative traces of mice in each group, as well as the quantification of the number of times they crossed the central area and the distance they moved in OFT test; **(D,G)** The representative trace heat maps of each group of mice and quantify the preference for new arms in Y-maze test; **(E,H)** The representative traces of mice in each group and quantifies the RI in NOR tests. n = 6, Means ± S.E.M. *P < 0.05, **P < 0.01 vs. the corresponding control. CCH, chronic cerebral hypoperfusion; OMT, oxymatrine; BCAS, bilateral carotid artery stenosis; NOR, novel objection recognition; OFT, open field test.

We first assessed the hemodynamic changes in BCAS mice, as shown in Figure 1. Compared to the control group, the CCH mice exhibited reduced cerebral blood supply due to BCAS modeling (Figure 1A). Laser speckle flow imaging revealed a significant reduction in cerebral blood flow in the CCH group (*P* < 0.01, Figure 1B). Next, we evaluated the effects of OMT treatment on recognition and spatial memory in CCH mice using the OFT, NOR, and Y-maze tests. In the OFT, the number of crossings and the total distance traveled in the central area were significantly lower in CCH mice compared to controls, but high-dose OMT reversed these deficits (*P* < 0.01, *P* < 0.05, Figures 1C,F). In the Y-maze test, the preference index, defined as the ratio of time spent exploring the new arm to the total time spent exploring both the new and familiar arms, was lower in the CCH group. However, OMT treatment significantly increased the preference index (*P* < 0.01, *P* < 0.05, Figures 1D,G). In NOR testing, the Recognition Index (RI), calculated as the ratio of time spent exploring a new object to the total time spent exploring both the new and familiar objects, was significantly reduced in CCH mice, indicating impaired memory function. In contrast, OMT treatment at various doses reversed this decline, leading to a significant increase in RI (*P* < 0.01, *P* < 0.05, Figures 1E,H). In summary, OMT effectively ameliorated the impairment in recognition and spatial memory in CCH mice.

### 3.2 OMT reversed the neurons damage and mitochondrial quality reduction in the hippocampal of CCH mice

**FIGURE 2:**
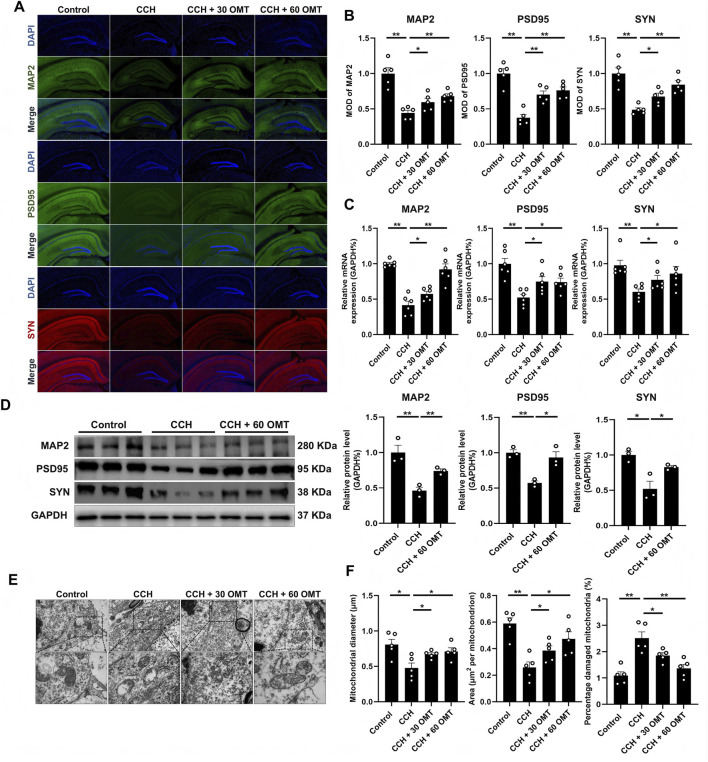
OMT reversed the neurons damage and mitophagy reduction in the hippocampal of CCH mice. **(A,B)** Representative immunofluorescence images and quantitative analysis of MAP2, PSD95 and SYN, n = 5; **(C)** The mRNA expression of MAP2, PSD95 and SYN, n = 6; **(D)** The protein expression and quantitative analysis of MAP2, PSD95 and SYN, n = 3; **(E,F)** The mass of mitochondria and the number of mitophagosomes observed by electron microscopy, n = 6. Means ± S.E.M. *P < 0.05, **P < 0.01 vs. the corresponding control. CCH, chronic cerebral hypoperfusion; OMT, oxymatrine; MAP2, microtubule-associated protein 2; PSD95, postsynaptic density protein-95; SYN, synaptophysin.

To further investigate the effects of OMT on hippocampal neuron function in CCH mice, we first measured the level of microtubule-associated protein 2 (MAP2), a marker that maintains neuronal morphology and stability. As shown in Figure 2A, MAP2 expression was significantly decreased in the hippocampal region of CCH mice compared to controls (*P* < 0.01, *P* < 0.05, Figures 2A,B). Synaptic plasticity, regulated by postsynaptic density protein-95 (PSD-95) and presynaptic synaptophysin (SYN), was also assessed by immunofluorescence. We observed lower levels of PSD-95 and SYN in the CCH group, while OMT treatment at various doses restored these synaptic proteins, mitigating neuronal damage in the hippocampus (*P* < 0.01, *P* < 0.05, Figures 2A,B). Consistent with these findings, mRNA expression of MAP2, PSD95, and SYN also showed significant recovery with OMT treatment (*P* < 0.01, *P* < 0.05, Figure 2C). At the protein level, high-dose OMT (60 mg/kg) exhibited the most pronounced therapeutic effect, significantly increasing MAP2, PSD95, and SYN expression compared to the CCH group (*P* < 0.01, *P* < 0.05, Figure 2D). Additionally, transmission electron microscopy revealed severe mitochondrial ultrastructural damage in the CCH group, including mitochondrial matrix expansion, reduced diameter, decreased area, and excessive damage. Mitochondrial autophagosomes were also fewer in number (*P* < 0.01, Figures 2E,F). These results suggest that damaged mitochondria accumulate in the hippocampus of CCH mice, with a reduced mitophagy function. However, OMT treatment reversed these mitochondrial and neuronal damages (*P* < 0.01, *P* < 0.05, Figures 2E,F). In conclusion, OMT ameliorated neuronal damage and mitophagy reduction in the hippocampus of CCH mice.

### 3.3 OMT restored the mitophagy reduction and the SIRT1 inhibition in the hippocampus of CCH mice

**FIGURE 3:**
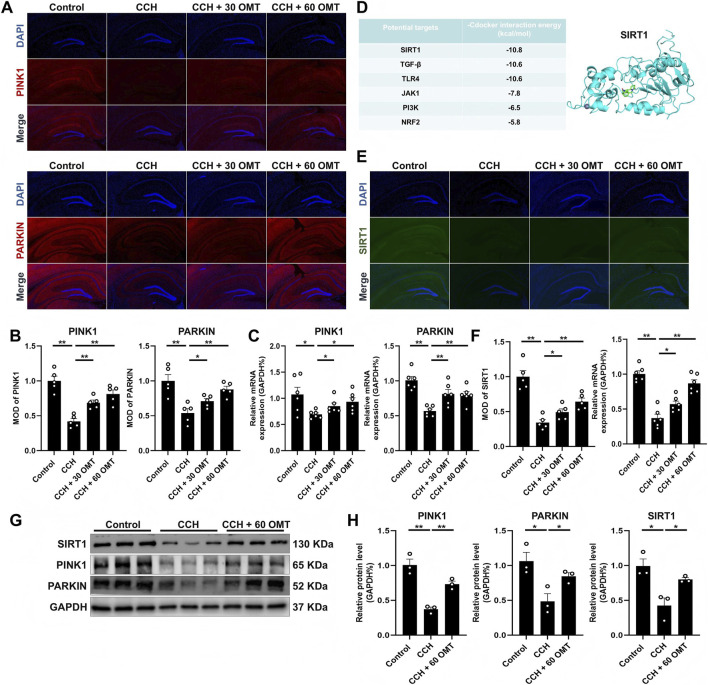
OMT restored the mitophagy reduction and the SIRT1 inhibition in the hippocampus of CCH mice. **(A,B)** Representative immunofluorescence images and quantitative analysis of PINK1and PARKIN, n = 5; **(C)** The mRNA expression of PINK1and PARKIN, n = 6; **(D)** Binding energy of OMT with downstream targets and binding sites of OMT to SIRT1; **(E,F)** Representative immunofluorescence images and quantitative analysis (n = 5), mRNA expression (n = 6) of SIRT1; **(G,H)** The protein expression and quantitative analysis of PINK1, PARKIN and SIRT1, n = 3. Means ± S.E.M. *P < 0.05, **P < 0.01 vs. the corresponding control. CCH, chronic cerebral hypoperfusion; OMT, oxymatrine; PINK1, PTEN-induced kinase 1; PARKIN, parkinson disease protein 2; SIRT1, Sirtuin 1.

We next investigated the effects of OMT on mitophagy in the hippocampus of CCH mice and explored its potential mechanisms. Initially, we examined the expression of mitophagy-related genes, PINK1 and PARKIN. Both immunofluorescence and mRNA analysis revealed a significant reduction in PINK1 and PARKIN expression in the hippocampus of CCH mice compared to controls. OMT treatment restored the expression of these mitophagy genes (*P* < 0.01, *P* < 0.05, Figures 3A–C). To explore the underlying mechanisms, we used Autodock software to analyze the interaction between OMT and its known downstream targets (Lu et al., 2016). The results indicated that Sirtuin 1 (SIRT1) binds to OMT with the lowest binding energy (Figure 3D). We then examined SIRT1 expression at the tissue level. CCH significantly decreased both the protein and mRNA levels of SIRT1 in the hippocampus, but OMT treatment reversed these effects (*P* < 0.01, *P* < 0.05, Figures 3E,F). Furthermore, CCH reduced the protein expression of PINK1, PARKIN, and SIRT1 in hippocampal tissue, while high-dose OMT (60 mg/kg) significantly increased their expression (*P* < 0.01, *P* < 0.05, Figures 3G,H). In conclusion, OMT restores mitophagy and SIRT1 expression in the hippocampus of CCH mice.

### 3.4 OMT reversed the OGD/R induced HT22 cell dysfunction, mitophagy inhibition and decreased SIRT1 expression

**FIGURE 4:**
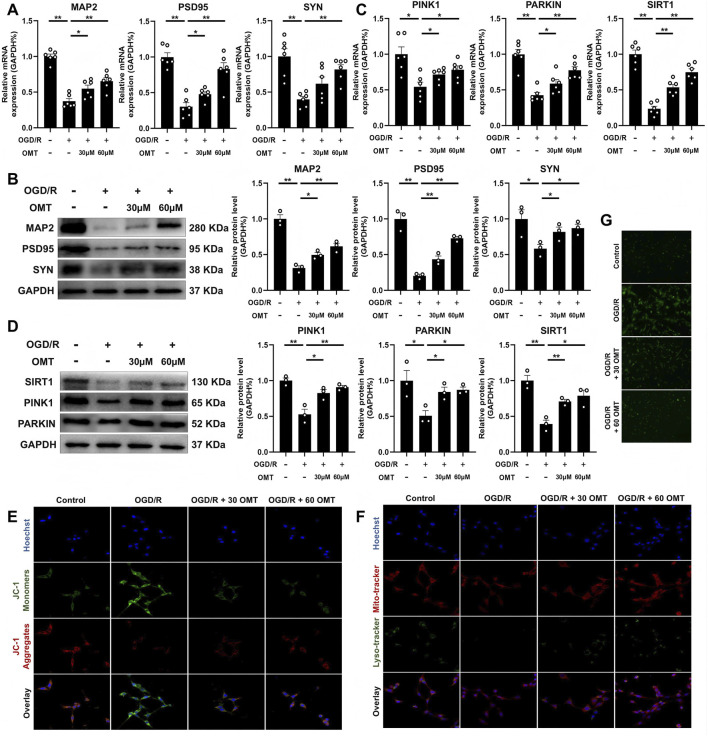
OMT reversed the OGD/R induced HT22 cell dysfunction, mitophagy inhibition and decreased SIRT1 expression. **(A)** The mRNA expression of MAP2, PSD95 and SYN, n = 6; **(B)** The protein expression and quantitative analysis of MAP2, PSD95 and SYN, n = 3; **(C)** The mRNA expression of PINK1, PARKIN and SIRT1, n = 6; **(D)** The protein expression and quantitative analysis of PINK1, PARKIN and SIRT1, n = 3; **(E)** Detection of JC-1 aggregates (red) and monomers (green) by confocal fluorescence microscopy, 1000×, n = 3; **(F)** Colocalization of lysosome (green) and mitochondria (red) by confocal fluorescence microscopy, 400×, 1000×, n = 3; **(G)** Representative ROS images, 100×, n = 5. Means ± S.E.M. *P < 0.05, **P < 0.01 vs. the corresponding control. CCH, chronic cerebral hypoperfusion; OMT, oxymatrine; MAP2, microtubule-associated protein 2; PSD95, postsynaptic density protein-95; SYN, synaptophysin; PINK1, PTEN-induced kinase 1; PARKIN, parkinson disease protein 2; SIRT1, Sirtuin 1.

To further confirm that OMT’s protective effects on neuronal function are mediated through the SIRT1/PINK1 pathway, we exposed HT-22 cells to an OGD/R environment and treated them with OMT. We observed that OGD/R significantly reduced the mRNA and protein expression of MAP2, PSD95, and SYN in HT-22 cells. However, treatment with various concentrations of OMT restored the expression of these proteins compared to the OGD/R group (*P* < 0.01, *P* < 0.05, Figures 4A,B). Mechanistically, OGD/R also inhibited the mRNA and protein levels of SIRT1, PINK1, and PARKIN, but OMT treatment reversed these changes (*P* < 0.01, *P* < 0.05, Figures 4C,D). To assess mitochondrial function, we used JC-1 probes, which aggregate at high mitochondrial membrane potential and exist as monomers at low potential. Immunofluorescence results showed that OGD/R reduced the number of JC-1 polymers (red) and increased mitochondrial monomers (green). In contrast, OMT treatment at different concentrations increased the number of polymers and decreased monomers (Figure 4E). Additionally, LysoTracker and MitoTracker staining revealed that OGD/R inhibited the co-localization of lysosomes and mitochondria, indicating impaired mitophagy. OMT treatment restored this co-localization (Figure 4F). Finally, the ROS staining indicated that OGD/R increased the intracellular reactive oxygen species levels, while OMT treatment could reduce ROS (Figure 4G). In conclusion, OMT reversed OGD/R-induced dysfunction in HT-22 cells, mitigated mitophagy inhibition, and restored SIRT1 expression.

### 3.5 SIRT1 and mitophagy mediate the protective effect of OMT on OGD/R HT22 cells

**FIGURE 5:**
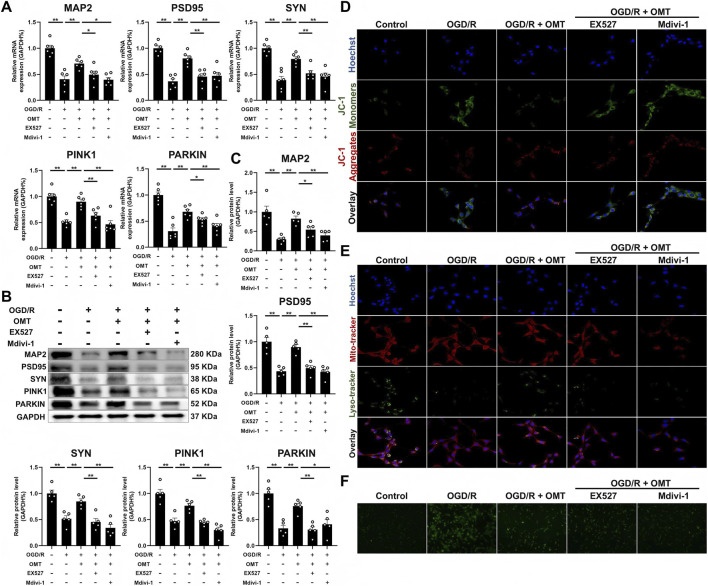
SIRT1 and mitophagy mediate the protective effect of OMT on OGD/R HT22 cells. **(A)** The mRNA expression of MAP2, PSD95, SYN PINK1 and PARKIN, n = 6; **(B,C)** The protein expression and quantitative analysis of MAP2, PSD95, SYN PINK1 and PARKIN, n = 3; **(D)** Detection of JC-1 aggregates (red) and monomers (green) by confocal fluorescence microscopy, 1000×, n = 3; **(E)** Colocalization of lysosome (green) and mitochondria (red) by confocal fluorescence microscopy, 400×, 1000×, n = 3; **(F)** Representative ROS images, 100×, n = 5. Means ± S.E.M. *P < 0.05, **P < 0.01 vs. the corresponding control. CCH, chronic cerebral hypoperfusion; OMT, oxymatrine; MAP2, microtubule-associated protein 2; PSD95, postsynaptic density protein-95; SYN, synaptophysin; PINK1, PTEN-induced kinase 1; PARKIN, parkinson disease protein 2; SIRT1, Sirtuin 1.

To further confirm whether OMT’s protective effects on HT-22 cells depend on the SIRT1/PINK1 pathway, we used the SIRT1 inhibitor EX527 and the mitophagy inhibitor Mdivi-1 and assessed relevant indicators. As shown in Figure 5, OMT restored the inhibitory effects of OGD/R on MAP2, PSD95, SYN, PINK1, PARKIN, and SIRT1 expression. However, the therapeutic effects of OMT were blocked by EX527 and Mdivi-1 treatment (*P* < 0.01, *P* < 0.05, Figure 5A). These changes were consistent at both the protein and mRNA levels (*P* < 0.01, *P* < 0.05, Figures 5B,C). JC-1 fluorescence results showed that OMT reversed the OGD/R-induced reduction in mitochondrial polymers and increase in monomers. However, EX527 and Mdivi-1 treatment prevented this reversal, with a continued reduction in polymers and an increase in monomers (Figure 5D). LysoTracker and MitoTracker staining further confirmed that OMT mitigated OGD/R-induced mitophagy inhibition, while EX527 and Mdivi-1 reversed these effects (Figure 5E). Furthermore, ROS staining revealed that OMT was able to alleviate the increase ROS level caused by OGD/R, and this phenomenon could also be reversed by EX527 and Mdivi-1 (Figure 5F). These findings indicate that SIRT1 and mitophagy mediate the protective effect of OMT on HT-22 cells under OGD/R conditions.

### 3.6 SIRT1 mediates the neuroprotective effect and mitophagy of OMT on the hippocampus of CCH mice

**FIGURE 6:**
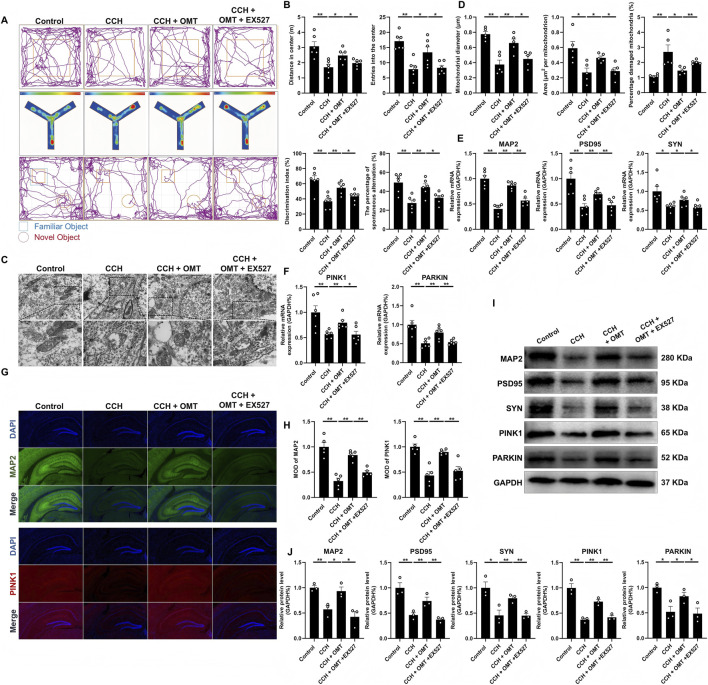
SIRT1 mediates the neuroprotective effect and mitophagy of OMT on the hippocampus of CCH mice. **(A,B)** The representative traces and quantitative analysis in OFT, Y-maze and NOR test, n = 6; **(C,D)** The mass of mitochondria and the number of mitophagosomes observed by electron microscopy, n = 6; **(E,F)** The mRNA expression of MAP2, PSD95, SYN PINK1 and PARKIN, n = 6; **(G,H)** Representative immunofluorescence images and quantitative analysis of MAP2 and PINK1, n = 5; **(I,J)** The protein expression and quantitative analysis of MAP2, PSD95, SYN PINK1 and PARKIN, n = 3. Means ± S.E.M. *P < 0.05, **P < 0.01 vs. the corresponding control. CCH, chronic cerebral hypoperfusion; OMT, oxymatrine; MAP2, microtubule-associated protein 2; PSD95, postsynaptic density protein-95; SYN, synaptophysin; PINK1, PTEN-induced kinase 1; PARKIN, parkinson disease protein 2; SIRT1, Sirtuin 1.

Finally, we verified at the *in vivo* level that SIRT1 mediates the neuroprotective effects of OMT on the hippocampus of CCH mice. Mice were treated with CCH and/or EX527, and relevant indicators were assessed in the hippocampal region. As shown in Figure 6, in the OFT, CCH mice exhibited significantly reduced total movement distance and fewer crossings in the central region compared to controls, but OMT treatment reversed these effects. In contrast, the OMT + EX527 group showed significantly reduced movement and central region crossings compared to the OMT group (*P* < 0.01, *P* < 0.05, Figures 6A,B). In the Y-maze test, the CCH group had a decreased preference index, which was restored by OMT. However, EX527 treatment reversed this effect (*P* < 0.01, *P* < 0.05, Figures 6A,B). Similarly, EX527 reduced the increase in the RI in CCH mice caused by OMT in the NOR test (*P* < 0.01, *P* < 0.05, Figures 6A,B). Transmission electron microscopy revealed that OMT restored CCH-induced mitochondrial damage and mitophagy dysfunction in hippocampal neurons, whereas EX527 weakened OMT’s therapeutic effects (*P* < 0.01, *P* < 0.05, Figures 6C,D). These results suggest that mitochondrial damage accumulates in the hippocampus of CCH mice and that mitophagy is impaired. OMT treatment increased the expression of genes related to neuronal function (MAP2, PSD95, SYN) and mitophagy (PINK1, PARKIN) that were reduced by CCH, while EX527 reduced the expression of these genes (*P* < 0.01, *P* < 0.05, Figures 6E,F). Immunofluorescence further confirmed that EX527 reversed OMT’s effects on MAP2 and PINK1 expression in CCH mice (*P* < 0.01, *P* < 0.05, Figures 6G,H). Protein expression levels of MAP2, PSD95, SYN, PINK1, and PARKIN mirrored their mRNA expression patterns (*P* < 0.01, *P* < 0.05, Figures 6I,J). These findings confirm that SIRT1 mediates the neuroprotective effects and mitophagy regulation of OMT in the hippocampus of CCH mice.

## 4 Discussion

Vascular cognitive dysfunction encompasses a spectrum of syndromes, ranging from mild cognitive dysfunction to dementia, caused by vascular risk factors and cerebrovascular diseases. CCH has been identified as a key pathophysiological mechanism underlying these conditions (Litak et al., 2020). Cohort studies have linked both sporadic and hereditary cerebral small vessel diseases to cerebral hypoperfusion (Iadecola et al., 2019). Risk factors for CCH, such as hypertension, type 2 diabetes, and atherosclerosis, can impair cerebral blood flow, leading to chronic insufficiency in cerebral perfusion. Over time, this damages white and gray matter, disrupting brain structure and function, and ultimately causing cognitive dysfunction (Chojdak-Lukasiewicz et al., 2021). Effective treatment for CCH remains a significant global challenge. Growing evidence suggests that oxymatrine (OMT) holds considerable potential for drug development, demonstrating therapeutic efficacy without toxic side effects (Huan et al., 2023). This study aims to investigate whether OMT can alleviate cognitive dysfunction caused by CCH, using the established BCAS mouse model and OGD/R cell model, which were widely recognized model for studying CCH-induced cognitive dysfunction (Tabassum et al., 2025; Pang et al., 2024). Our findings show that CCH leads to decreased cerebral blood volume and cognitive dysfunction in mice, specifically impairing recognition and spatial memory. OMT treatment reversed these cognitive deficits, and also mitigated neuronal dysfunction in hippocampal cells following OGD/R. These results suggest that OMT may offer a promising therapeutic strategy for alleviating cognitive dysfunction in CCH patients. The cholinergic system is a critical regulator of cognition.

OMT exhibits extensive neuroprotective potential in experimental neurological diseases (Dhurandhar et al., 2024). Pharmacokinetic data indicate that OMT is absorbed relatively quickly after oral administration (oral bioavailability in rats is 26.43%, peak time is 0.75 h, and half-life is 4.181 h), and its tissue distribution is mainly concentrated in the intestine, stomach, liver, and spleen (Wang et al., 2022). Additionally, experimental studies have shown that OMT can reduce the permeability of the blood-brain barrier and maintain tight junction function after cerebral ischemia-reperfusion, which supports the neurovascular protective effect (Jiao-Yan et al., 2021). The existing human data for OMT mainly come from non-neurological diseases: randomized and clinical studies in chronic hepatitis B and psoriasis have shown that its short-term safety and clinical activity are acceptable (Shi et al., 2024; Shi et al., 2019; Lu et al., 2003). Therefore, the human therapeutic dose, long-term safety, and translational applicability of OMT in vascular cognitive dysfunction still need to be further determined. Moreover, we did not include positive control drugs such as donepezil or memantine (Hershey and Coleman-Jackson, 2019). This is a limitation of this study. Future research should include direct comparisons with cognitive enhancers used in clinical practice and evaluate whether OMT also affects cholinergic signaling.

Mitochondria and their associated signaling pathways regulate synaptic signaling and mediate long-term structural and functional changes in neurons. Protein turnover and mitophagy are crucial for neuroprotection, helping maintain mitochondrial integrity and function (Wang et al., 2023). Mitophagy is an evolved cellular process that removes damaged mitochondria, ensuring metabolic balance, energy supply, neuronal survival, and overall neuronal health (Mani et al., 2021). In the hippocampus, mitophagy contributes to synaptic formation, axon development, dopamine release, and the improvement of long-term depression. In contrast, deficiencies in mitophagy lead to the accumulation of damaged mitochondria, which can impair cellular function, contributing to aging and neurodegenerative diseases (Cen et al., 2021). Therefore, mitophagy likely plays a pivotal role in the neuronal damage observed in CCH mice induced by BCAS. Our study found that CCH induced significant mitochondrial swelling, vacuolar degeneration, and a decrease in mitochondrial autophagosomes. However, OMT treatment alleviated these changes. In HT22 cells, OMT also reversed mitochondrial dysfunction and reduced autophagosome formation caused by OGD/R. These findings suggest that OMT effectively promotes mitophagy both *in vivo* and *in vitro*, thereby restoring neuronal function.

It is noteworthy, however, that previous studies have shown that CCH and OGD/R increase mitophagy and induce neuronal dysfunction, which contrasts with our results. Nevertheless, all these damages can be reversed by promoting mitophagy, which aligns with our conclusions (Fang et al., 2025; Mao et al., 2022; Yang et al., 2024; Zhao et al., 2024). One possible explanation is that mitophagy responses may be highly dependent on the severity, duration, and temporal profile of cerebral hypoperfusion, as well as on the specific experimental model used. In many earlier animal studies, chronic cerebral hypoperfusion was induced by bilateral common carotid artery occlusion (BCCAo), which causes a rapid and profound reduction in cerebral blood flow. Zhou’s study demonstrated that BCCAo led to a 90% reduction in blood flow within a short period, causing immediate spatial memory impairments. In contrast, the BCAS model induces a long-term 30% reduction in cerebral blood flow, leading to significant memory impairments over time (Zhou et al., 2022). The BCAS model is generally considered to produce a more gradual and sustained hypoperfusion state, and has been widely used as a model of subcortical ischemic vascular cognitive dysfunction (Ishikawa et al., 2023). Based on these differences, we speculate that mitophagy may exhibit a dynamic and context-dependent pattern. Under acute or severe ischemic stress, mitophagy may be transiently activated as an adaptive response to eliminate damaged mitochondria. However, under prolonged and relatively moderate hypoperfusion, persistent mitochondrial injury, impaired bioenergetics, and disruption of autophagic flux may eventually lead to insufficient or dysfunctional mitophagy, rather than sustained activation (Yu et al., 2023; He et al., 2024). *In vitro*, culturing for less than 4 h under sugar-free and oxygen-free conditions can lead to an increase in HT22 mitophagy levels (Mao et al., 2022; Yang et al., 2024), while culturing for more than 6 h will inhibit mitophagy (Chen et al., 2025; Shao et al., 2021). Compared with the current symptomatic treatment methods, inducing mitophagy may represent a mechanism-based therapeutic strategy, as it directly targets mitochondrial quality control, which is a key link in the critical pathological process of chronic cerebral hypoperfusion. By promoting the clearance of damaged mitochondria, mitophagy may reduce oxidative stress, protect synaptic function and increase neuronal survival rate. In contrast, the currently available cognitive enhancers mainly provide symptomatic benefits and do not directly address the issue of mitochondrial dysfunction (Lyu et al., 2024). Therefore, inducing mitophagy may have potential value in improving the disease. However, the clinical translation of targeted therapy targeting mitochondria is still at an early stage, and further clinical studies are needed to evaluate its safety and efficacy.

SIRT1, an NAD + -dependent deacetylase, plays a crucial role in regulating various pathophysiological processes (Ding et al., 2024), and its involvement in autophagy is widely recognized as a cellular protective mechanism (Tang et al., 2022). Studies have shown that nicotinamide extends the replication lifespan of primary human fibroblasts by enhancing mitophagy, an effect mediated by an increased NAD+/NADH ratio and SIRT1 activation (Jang et al., 2012). Additionally, the SIRT1 activator resveratrol exerts protective effects on the heart through a signaling axis involving SIRT1-SIRT3 and PINK1/Parkin-mediated mitophagy. Conversely, the absence of SIRT1 in human prostate cancer cells delays Parkin translocation to mitochondria, reducing mitophagy (Tang, 2016). In this study, molecular docking suggested that OMT might target SIRT1 to exert its effects. Both animal and cell experiments confirmed that OMT reversed CCH- or OGD/R-induced inhibition of the PINK1/Parkin pathway and neuronal function. Although pharmacological inhibition with EX527 suggested that SIRT1 is involved in OMT-mediated activation of mitophagy, this evidence does not fully establish a direct causal relationship. Future studies using SIRT1 knockdown, knockout, or other genetic approaches will be required to further confirm that SIRT1 directly regulates PINK1/Parkin-mediated mitophagy in CCH. Moreover, the SIRT1 inhibitor EX527 abolished the protective effects of OMT on mitophagy and neuronal dysfunction.

**FIGURE 7:**
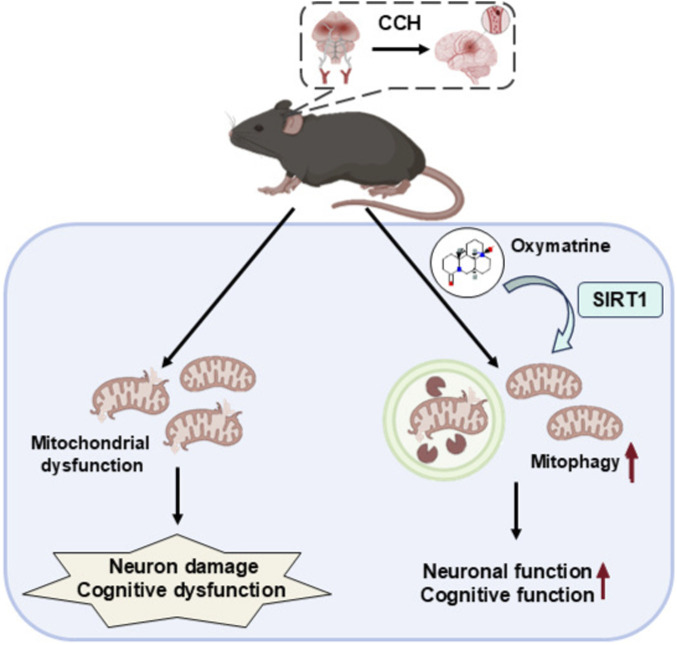
Oxymatrine alleviates cognitive dysfunction in mice caused by chronic cerebral hypoperfusion by promoting SIRT1/ PINK1-mediated mitophagy. CCH, chronic cerebral hypoperfusion; SIRT1, Sirtuin 1.

In conclusion, OMT enhances mitophagy and mitigates neuronal dysfunction and cognitive dysfunction induced by CCH or OGD/R by targeting SIRT1. This study suggests that OMT may serve as a potential therapeutic agent for senile dementia (Figure 7).

## Data Availability

The raw data supporting the conclusions of this article will be made available by the authors, without undue reservation.
